# Identification of reliable reference genes for qRT-PCR in the ephemeral plant *Arabidopsis pumila* based on full-length transcriptome data

**DOI:** 10.1038/s41598-019-44849-1

**Published:** 2019-06-10

**Authors:** Yuhuan Jin, Fang Liu, Wei Huang, Qi Sun, Xianzhong Huang

**Affiliations:** 1 Special Plant Genomics Laboratory, College of Life Sciences, Shihezi University, Shihezi, Xinjiang 832000 China; 2 State Key Laboratory of Plant Physiology and Biochemistry, College of Biological Sciences, China Agricultural University, Beijing, 100193 China

**Keywords:** Reverse transcription polymerase chain reaction, Plant sciences

## Abstract

*Arabidopsis pumila*, an annual ephemeral plant, plays important roles in preventing wind and sand erosion, water and soil conservation, and microhabitat improvement in the North of Xinjiang, China. Studies of adaptive mechanisms in harsh desert environments at the genetic and genomic levels can be used to more effectively develop and protect this species. The quantitative real-time polymerase chain reaction (qRT-PCR) method is one of the essential means to achieve these goals, and the selection of an appropriate reference gene is the prerequisite for qRT-PCR. In this study, 10 candidate reference genes were identified from the full-length transcriptome data of *A. pumila*, and their expression stabilities under four abiotic stresses (drought, heat, cold and salt) and in seven different tissues (roots, hypocotyl, cotyledon, leaves, stems, flowers and siliques) were evaluated with four programmes geNorm, NormFinder, Bestkeeper and RefFinder. Although the most stable reference genes were variable under different treatments using different software, comprehensive ranking revealed that *UEP* and *HAF1* under drought stress, *UBQ9* and *GAPDH* under heat stress, *UBC35* and *GAPDH* under cold stress, *GAPDH* and *ACT1* under salt stress, and *ACT1* and *GAPDH* in different tissues were the most stable reference genes. Moreover, *GAPDH* and *UBQ9* were the most suitable reference gene combinations for all samples. The expression pattern of the K^+^ uptake permease gene *KUP9* further validated that the selected reference genes were suitable for normalization of gene expression. The identification of reliable reference genes guarantees more accurate qRT-PCR quantification for *A. pumila* and facilitates functional genomics studies of ephemeral plants.

## Introduction

Ephemeral comes from Greek “ephémeros”, which means transitory and quickly fading, lasting only one day. In botany, an ephemeral plant is a short-lived plant that has one or more generations per year, growing only during favourable periods (e.g., when adequate water is available), and surviving the unfavourable periods in the form of seeds^1^. In China, ephemeral plants are mainly distributed in the desert environments of the Junggar Basin and the Yili River valley in northern Xinjiang, with the eastern margin of the Junggar Basin as the boundary. Ephemeral plants sprout in mid-March or early April each year, making use of rain and snow water melt from Tianshan Mountain, and complete their life-cycle within approximately two months by mid-June, a period with the strongest wind force in the desert regions of northern Xinjiang^1,2^. Accordingly, ephemeral plants play important roles as windbreaks and in the stabilization of sand, water and soil conservation, and microhabitat improvement. *Arabidopsis pumila* (synonym: *Olimarabidopsis pumila*), an ephemeral plant, is well adapted to take advantage of the short favourable seasons in desert areas (Supplementary Fig. S1)^2,3^. In addition to fast growth, it also has the advantages of high yield, high photosynthetic efficiency and stress tolerance^3,4^. To utilize and protect this ephemeral plant, it is of great significance to elucidate the molecular mechanism of rapid growth and adversity adaptation. Based on the above reasons, our group generated a full-length transcriptome database of *A. pumila* seedlings using a combination of single-molecule real-time (SMRT) sequencing and Illumina sequencing^3^.

Quantitative real-time polymerase chain reaction (qRT-PCR) is a commonly used and powerful tool for accurately analysing gene expression levels in different samples or under different experimental conditions in a wide range of biological experiments^5^. Compared with traditional quantitative analysis techniques of gene expression, qRT-PCR has the advantages of simplicity, precision, specificity, sensitivity, flexibility, scalability and high throughput capacity^5–8^. It is well known that the appropriate choice of reference genes for normalizing expression levels of a test gene is crucial to the accuracy and reliability of qRT-PCR data. The ideal reference genes should have a moderate and stable expression level in different tissues at different developmental stages and under different experimental treatments^9–11^. Some of the most frequently used reference genes for qRT-PCR in animals and plants are 18S rRNA, actin (*ACT1* and *ACT2*), α-tubulin and β-tubulin (*TUA* and *TUB*), cyclophilin (*CYP1* and *CYP2*), elongation factor-1α (*EF-1α*), glyceraldehyde-3-phosphate dehydrogenase (*GAPDH*), polyubiquitin (*UBQ*), and ribosomal protein L (*RPL1* and *RPL2*)^9,12–17^. Unfortunately, several studies have shown that the transcripts of these reference genes are not always stable when subjected to various experimental conditions^18–20^. The screening of stable reference genes is now gradually attracting scientists’ attention. A wiki-driven database of internal control genes (ICG: http://icg.big.ac.cn) has been constructed for qRT-PCR normalization involving a wide range of specific tissues, development stages and experimental treatments. ICG has integrated 209 species, including 73 animals, 115 plants, 12 fungi and 9 bacteria, and has curated 757 internal control genes with validation under more than 70 types of experimental conditions, such as salinity, heat and insecticide treatment^21^.

However, no reference genes are available for transcript normalization in *A. pumila*, so we were unable to validate our transcriptome sequencing data, analyse the expression profiles of salt or other stress-related genes, or further reveal the adaptive growth mechanism of *A. pumila* to desertification. The objective of this study was to identify stable reference genes in *A. pumila* subjected to various abiotic stresses as well as different developmental tissues.

## Results

### Amplification specificity and amplification efficiency of the candidate reference genes and target genes

A total of 10 candidate reference genes and one target gene from the *A. pumila* full-length transcriptome database were selected for qRT-PCR normalization. The unigene name, gene symbol, homologue locus and *E* value compared with those of the homologous genes are summarized in Table 1. The primer sequences, amplicon size, product Tm, and amplification efficiencies are shown in Table 2. The size of PCR products for each reference gene was tested by 2.0% agarose gel, which provided the expected amplicon length (Supplementary Fig. S2). The specificity for each primer pair was verified by melting curve analysis, which revealed that each gene had a single amplification peak (Supplementary Fig. S3). qRT-PCR efficiency for all 10 candidate reference genes ranged from 90.3% for *EF1B* to 99.1% for *UEP*, and correlation coefficients varied from 0.988 (*ACT2*) to 0.999 (*LOS1*) (Table 2).

**Table 1:** Description of candidate reference genes and target genes.

Gene name	Gene symbol	Homolog locus	Gene ID	*E* value
Actin-1	*ACT1*	AT2G37620.1	2–3k.c16834/1/1406	0.0
Actin-2	*ACT2*	AT3G18780.2	2–3k.c6923/1/1569	0.0
Aldehyde dehydrogenase	*ALDH*	AT3G66658.1	1–2k.c31292/1/2035	0.0
Translation elongation factor	*EF1B*	AT5G19510.1	2–3k.c8091/1/833	e-168
Histone acetyltransferase	*HAF1*	AT1G32750.1	3–6k.c17817/1/4957	0.0
Glyceraldehyde-3-phosphate dehydrogenase	*GAPDH*	AT1G13440.1	2–3k.c18384/1/1353	0.0
Low expression of osmotically responsive genes 1	*LOS1*	AT1G56070.1	1–2k.c36904/1/2756	0.0
Polyubiquitin 9	*UBQ9*	AT5G37640.1	1–2k.c36785/1/1730.1	1e-61
Ubiquitin-conjugating enzyme 35	*UBC35*	AT1G78870.1	1–2k.c18460/1/1770.2	3e-84
Ubiquitin extension protein	*UEP*	AT3G52590.1	1–2k.c21768/1/663	0.0
K^+^ uptake permease 9	*KUP9*	AT4G19960.1	2–3k.c27754/1/2830	0.0

**Table 2:** Primers used in this study and their amplification efficiency and amplification characteristics.

Genes	Primer sequence (5′-3′)	Tm (°C)	R^2^	E (%)	Amplicon size (bp)
*ACT1*	F: CGACAATGGAACTGGAATGGTTA	59	0.997	0.936	296
	R: GTGCCTCGGTAAGTAGAATAGGA	59			
*ACT2*	F: TGTGCCAATCTACGAGGGTT	59	0.988	0.934	137
	R:TTTCCCGTTCTGCTGTTGTG	59			
*ALDH*	F: ACAACGGCACTACTGGATCA	59	0.994	0.920	224
	R: CCAATGCAAGCCTGTCCATT	59			
*EF1B*	F: GCAACAATGGCCGTTACCT	59	0.996	0.903	153
	R: ATCGCTGGGTTTCACTGGA	59			
*GAPDH*	F:CTCCCATGTTTGTTGTTGGTGTCA	62	0.997	0.951	217
	R: CTTCCACCTCTCCAGTCCTTCATT	62			
*HAF1*	F: TAAGTCCACGTCTAAGATCAGCA	59	0.997	0.938	186
	R: GCCTGGTTTCTCTCTGTACTAGA	59			
*LOS1*	F: GGCAAGAGACTGTGGAGGA	59	0.999	0.909	162
	R: CAGCAACACGAACAACAGGA	59			
*UBC35*	F: GAGGATCACGTCAGATTCGAG	58	0.998	0.907	163
	R: AGTTTCCTTGATGATTCTTCGCG	60			
*UBQ9*	F: GTGCTGAGAGATGCGGATTG	59	0.998	0.908	252
	R: CCTCTCCTCCTCCAACAGTC	59			
*UEP*	F: GAGGTCGAGAGCAGTGACAC	60	0.998	0.991	178
	R: CCCTGAGCCTCAACACAAGA	60			
*KUP9*	F: TGTTCTTTCGGCAACTGGTG	59	1.000	0.979	284
	R: AAATCCAACCATCCCGACCT	59			

### Expression profiles of the candidate reference genes

**Figure 1:**
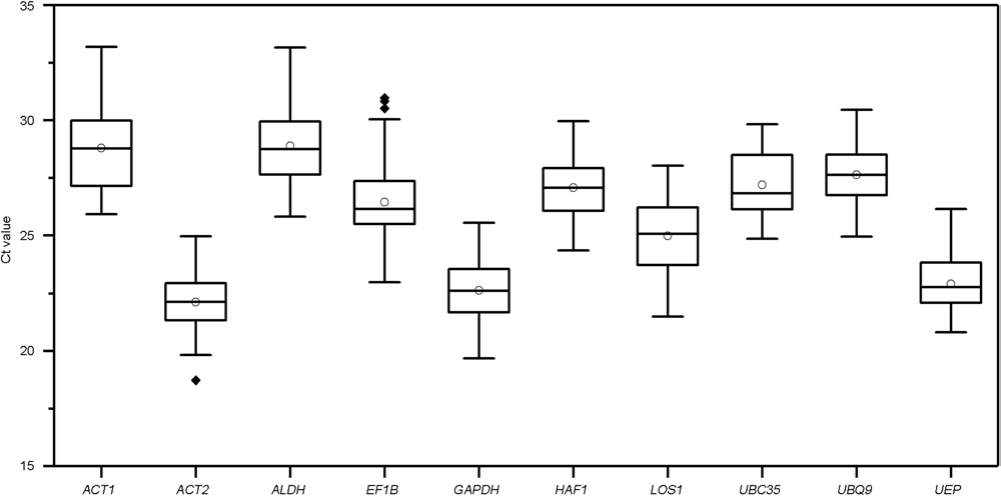
Distribution of Ct values for 10 candidate reference genes across all *A. pumila* samples using qRT-PCR data. The solid line within each box represents the median Ct values, and boxes denote the 25th and 75th percentiles. Circles and black dots represent the mean Ct values and potential outliers, respectively.

Boxplot analysis of Ct values of the 10 different reference genes in all experimental samples showed the means and standard deviation of the Ct values for each reference gene (Fig. 1). The results showed that the reference genes displayed a wide range of transcription levels across all test samples, with average Ct values ranging from 22.13 to 28.89 (Supplementary Table S1). Because gene expression levels are negatively correlated with Ct values, *ACT2* exhibited the highest expression abundance, with the lowest mean Ct values. Both *GAPDH* and *UEP* showed relatively high expression, with mean Ct values of 22.63 and 22.91, respectively, whereas *ACT1* and *ALDH* showed relatively low expression (mean Ct = 28.79 and 28.89). The expression levels of other genes were at the mean level.

### Analysis of expression stability of the candidate reference genes

Expression stability of the 10 reference genes was carried out using geNorm^22^, NormFinder^14^, BestKeeper^23^ and RefFinder^24,25^ software.

#### GeNorm analysis

**Figure 2:**
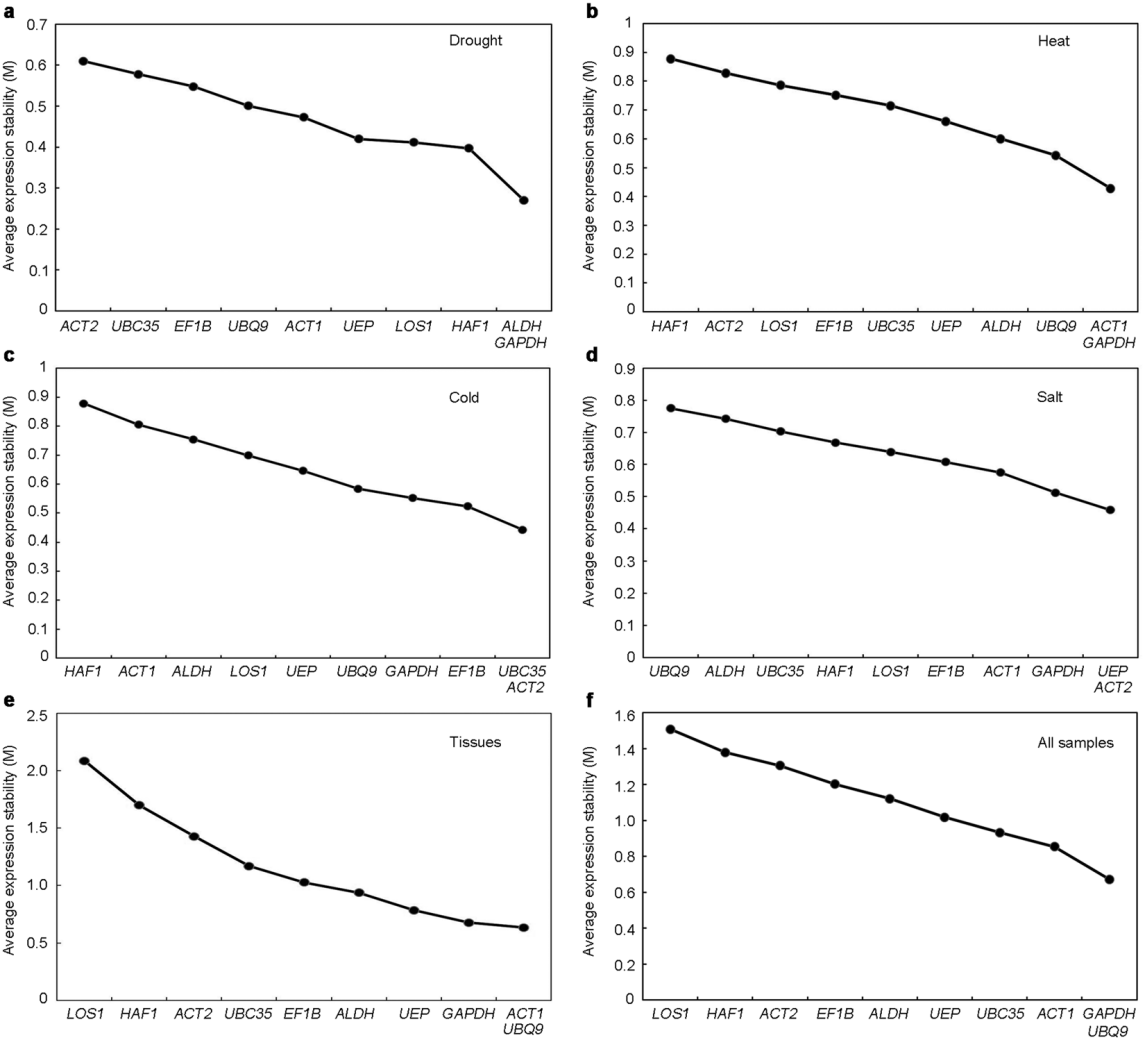
geNorm analysis of the stability values (M) of 10 candidate reference genes under different treatments and in different tissues. (**a**) Drought treatment. (**b**) Heat treatment. (**c**) Cold treatment. (**d**) Salt treatment. (**e**) Tissues. (**f**) All samples. The most stable genes are arranged on the right, while the least stable genes are on the left.

Gene expression stability was evaluated by the M value using geNorm analysis. The lower the M value, the higher the gene expression stability^22^. We first calculated the stable value M of the reference gene and sorted the M value from high to low (Fig. 2). We found that all 10 reference genes under abiotic stresses and in different tissues had M values below 1.5, suggesting that these genes were stable across all these experimental sets. *ALDH* and *GAPDH* under drought stress (10% PEG-6000), *ACT1* and *GAPDH* under heat stress (40 °C), and *ACT2* and *UBC35* under cold stress (4 °C) were the most stable reference genes, with the lowest M values of 0.250, 0.428 and 0.442, respectively (Fig. 2a–c). At the same time, *UEP* and *ACT2* were considered to be the most stable genes under the treatment of salt stress (250 mM NaCl) with an M value of 0.458 (Fig. 2d). In the experimental developmental tissue group, *ACT1* and *UBQ9* with the lowest M value of 0.635 were ranked as the most stable genes, while *LOS1* (2.085) was ranked as least stable (Fig. 2e). In addition, *GAPDH* and *UBQ9* were suggested to be the two most stable genes with the lowest M value of 0.673 across all samples (Fig. 2f). In contrast, *LOS1* was the least stable gene in all treatments, with the highest M value of 1.508.

**Figure 3:**
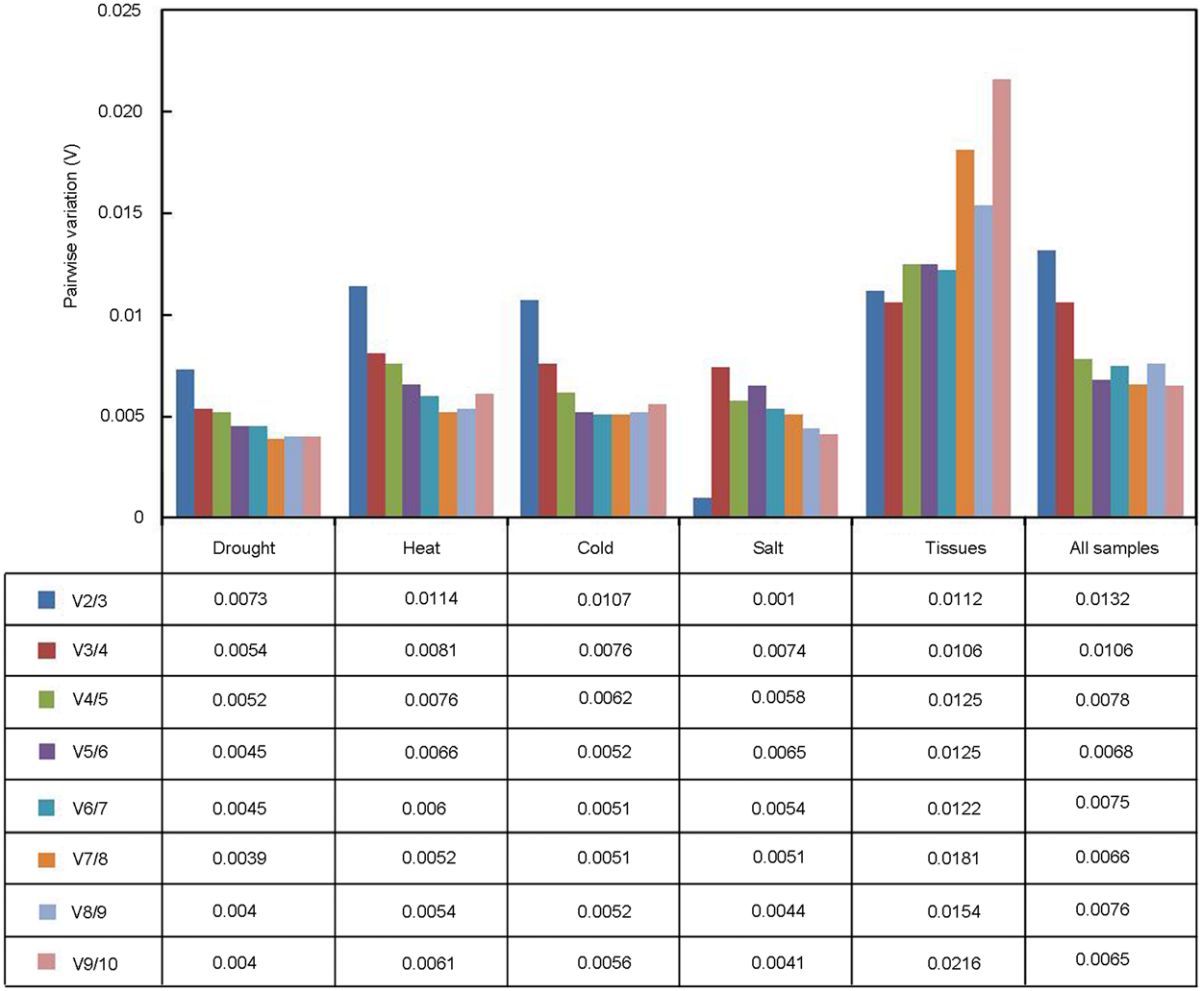
Optimal number of reference genes for normalization under the test experimental conditions. geNorm was used to calculate the pairwise variation (V_n_/V_n+1_, n represents reference gene numbers) to evaluate the number of reference genes in each experimental group.

To determine the optimal number of reference genes for qRT-PCR normalization, the pairwise variation value (V_n_/V_n+1_, n represents the reference gene number) between sequential normalization factors was also calculated by geNorm for all sample sets^22^. The results showed that the V_n_/V_n+1_ values for different experimental groups were below the cut-off value of 0.15 (Fig. 3), which indicated that two reference genes would be sufficient for normalization of qRT-PCR analysis in *A. pumila*.

#### NormFinder analysis

Expression stability values for each candidate reference gene were further analysed by NormFinder, which is based on variance analysis to calculate the stable value of each gene^14^. Similar to geNorm, a higher stable value indicates a more unstable gene, whereas a lower stable value indicates greater gene stability. According to the stability values calculated by NormFinder (Table 3), *UEP* (0.214) and *HAF1* (0.271) were the two most stable genes under drought stress conditions; *UBQ9* (0.196) and *GAPDH* (0.451) were the two most stable genes under heat treatment; *GAPDH* (0.269) and *EF1B* (0.291) were the two most stable genes under cold treatment; and *GAPDH* (0.162) and *ACT1* (0.380) were the two most stable genes in the salt stress group. Among different developmental tissues, *UBC35* and *ACT1* were the two most stable reference genes with the lowest stability values of 0.299 and 0.375, respectively. In addition, *GAPDH* and *UBQ9* were the most stable reference genes across all samples, with the lowest stability values of 0.479 and 0.601, respectively, and *LOS1* was the most unstable gene with the highest stability value of 1.777, which is completely consistent with the results determined through geNorm.

**Table 3:** Expression stability values (M) of 10 candidate reference genes calculated by NormFinder.

Rank	Drought	Heat	Cold	Salt	Tissues	All samples
1	*UEP*	*UBQ9*	*GAPDH*	*GAPDH*	*UBC35*	*GAPDH*
Stability	0.214	0.196	0.269	0.162	0.299	0.479
2	*HAF1*	*GAPDH*	*EF1B*	*ACT1*	*ACT1*	*UBQ9*
Stability	0.271	0.451	0.291	0.380	0.375	0.601
3	*LOS1*	*ALDH*	*UBC35*	*HAF1*	*UBQ9*	*UBC35*
Stability	0.323	0.463	0.295	0.439	0.442	0.670
4	*UBQ9*	*ACT1*	*ACT2*	*ACT2*	*GAPDH*	*UEP*
Stability	0.398	0.556	0.421	0.507	0.445	0.858
5	*ACT1*	*EF1B*	*UBQ9*	*UEP*	*ALDH*	*ACT1*
Stability	0.451	0.610	0.519	0.557	0.560	0.871
6	*ALDH*	*UEP*	*LOS1*	*LOS1*	*UEP*	*EF1B*
Stability	0.474	0.612	0.698	0.603	0.612	1.173
7	*EF1B*	*UBC35*	*UEP*	*UBC35*	*EF1B*	*ACT2*
Stability	0.490	0.673	0.725	0.640	0.617	1.253
8	*GAPDH*	*LOS1*	*ALDH*	*EF1B*	*ACT2*	*HAF1*
Stability	0.513	0.715	0.780	0.647	0.773	1.340
9	*UBC35*	*ACT2*	*ACT1*	*UBQ9*	*HAF1*	*ALDH*
Stability	0.529	0.868	0.917	0.729	0.911	1.344
10	*ACT2*	*HAF1*	*HAF1*	*ALDH*	*LOS1*	*LOS1*
Stability	0.629	0.922	1.047	0.735	1.492	1.777

#### BestKeeper analysis

We then used another programme, BestKeeper, to reanalyse the expression stability of the candidate reference genes. In our experiment, the r value was used to assess the stability of the reference genes. The higher the r value, the more stable the expression of the reference gene^23^. As shown in Table 4, under drought stress, *LOS1* (r = 0.983) and *UEP* (r = 0.980) were considered to be the two most stable reference genes, which is not consistent with geNorm and NormFinder. Under heat stress, *UBQ9* (r = 0.956) had the highest stability, which is consistent with the result of NormFinder. Under cold stress, *GAPDH* (r = 0.966) and *EF1B* (0.896) were the two most stable genes, which is completely consistent with the result of NormFinder. Similarly, the highest r value of 0.966 was observed under salt stress and for all developmental tissues, showing *GAPDH* and *UBC35* as the most stable reference genes, respectively. The most unstable genes determined by BestKeeper were also consistent with the results of NormFinder: *ACT2* under drought, *HAF1* in the heat and cold groups, *ALDH* under salt stress and *LOS1* in all tissues. In addition, *GAPDH* (r = 0.929) and *ACT1* (0.916) were the two most stable reference genes for all samples, and *LOS1* (r = 0.563) was the most unstable gene.

**Table 4:** Expression stability values (M) of 10 candidate reference genes calculated by BestKeeper.

Rank	Drought	Heat	Cold	Salt	Tissues	All samples
1	*LOS1*	*UBQ9*	*GAPDH*	*GAPDH*	*UBC35*	*GAPDH*
r value	0.983	0.956	0.966	0.966	0.966	0.929
2	*UEP*	*ACT1*	*EF1B*	*ACT1*	*UEP*	*ACT1*
r value	0.980	0.935	0.896	0.952	0.965	0.916
3	*HAF1*	*GAPDH*	*ALDH*	*LOS1*	*ACT1*	*UBQ9*
r value	0.975	0.910	0.895	0.934	0.933	0.909
4	*ACT1*	*EF1B*	*LOS1*	*UBQ9*	*UBQ9*	*UEP*
r value	0.971	0.901	0.873	0.919	0.921	0.901
5	*ALDH*	*ALDH*	*UBC35*	*EF1B*	*GAPDH*	*UBC35*
r value	0.955	0.882	0.869	0.916	0.910	0.892
6	*GAPDH*	*LOS1*	*ACT1*	*UEP*	*HAF1*	*HAF1*
r value	0.950	0.802	0.862	0.874	0.878	0.788
7	*UBQ9*	*UEP*	*ACT2*	*HAF1*	*ALDH*	*EF1B*
r value	0.947	0.792	0.837	0.866	0.853	0.782
8	*EF1B*	*ACT2*	*UBQ9*	*ACT2*	*EF1B*	*ALDH*
r value	0.925	0.775	0.746	0.846	0.833	0.751
9	*UBC35*	*UBC35*	*UEP*	*UBC35*	*ACT2*	*ACT2*
r value	0.908	0.752	0.584	0.730	0.746	0.748
10	*ACT2*	*HAF1*	*HAF1*	*ALDH*	*LOS1*	*LOS1*
r value	0.871	0.727	0.001	0.717	0.001	0.563

#### ΔCt analysis

According to the analysis of the ΔCt method calculated by RefFinder^24,25^ software (Supplementary Table S2), *UEP* and *HAF1* were the two most stable reference genes under drought stress, while *ACT2* was the most unstable gene, which is consistent with the analysis of NormFinder. *UBQ9* under heat stress, *GAPDH* under cold stress, and *GAPDH* under salt stress were the most stable genes, which is also completely consistent with the NormFinder and Bestkeeper analyses. In addition, the most stable reference gene in different tissues was *ACT1*, which is consistent with geNorm analysis but not with NormFinder and Bestkeeper; the most unstable gene was *LOS1*, which is consistent with the analysis determined using the three other programmes.

#### Comprehensive ranking of the candidate reference genes

The four different programmes yielded the same or divergent ranking for the stable reference genes, and this variation has also been reported in several studies^26–30^. To rank the reference genes under different stress conditions and in different experimental tissues, we further carried out comprehensive stability analysis for all 10 candidate reference genes. The final ranking result of expression stability for all reference genes was sorted according to the geometric mean of the reference genes (Table 5)^22,26^. According to the analysis results, *UEP* and *HAF1*, *UBQ9* and *GAPDH*, *UBC35* and *GAPDH* were the most stable genes under drought stress, heat stress and cold stress, respectively. The most stable reference genes under salt stress and in different tissues were both *GAPDH* and *ACT1*. Under all of the experimental conditions, *GAPDH* and *UBQ9* were the most stable reference genes, and *LOS1* was the most unstable reference gene.

**Table 5:** Ranking of expression stability for the 10 candidate reference genes.

Methods	1	2	3	4	5	6	7	8	9	10
**A. Drought stress (Ranking order from better-good-average)**										
geNorm	*GAPDH/ALDH*	*HAF1*	*LOS1*	*UEP*	*ACT1*	*UBQ9*	*EF1B*	*UBC35*	*ACT2*	
NormFinder	*UEP*	*HAF1*	*LOS1*	*UBQ9*	*ACT1*	*ALDH*	*EF1B*	*GAPDH*	*UBC35*	*ACT2*
Bestkeeper	*LOS1*	*UEP*	*HAF1*	*ACT1*	*ALDH*	*GAPDH*	*UBQ9*	*EF1B*	*UBC35*	*ACT2*
∆CT	*UEP*	*HAF1*	*LOS1*	*UBQ9*	*ALDH*	*ACT1*	*GAPDH*	*EF1B*	*UBC35*	*ACT2*
Comprehensive	*UEP*	*HAF1*	*UBQ9*	*LOS1*	*ALDH*	*GAPDH*	*UBC35*	*ACT1*	*EF1B*	*ACT2*
**B. Heat stress (Ranking order from better-good-average)**										
geNorm	*ACT1/GAPDH*	*UBQ9*	*ALDH*	*UEP*	*UBC35*	*EF1B*	*LOS1*	*ACT2*	*HAF1*	
NormFinder	*UBQ9*	*GAPDH*	*ALDH*	*ACT1*	*EF1B*	*UEP*	*UBC35*	*LOS1*	*ACT2*	*HAF1*
Bestkeeper	*UBQ9*	*ACT1*	*GAPDH*	*EF1B*	*ALDH*	*LOS1*	*UEP*	*ACT2*	*UBC35*	*HAF1*
∆CT	*UBQ9*	*GAPDH*	*ALDH*	*ACT1*	*UEP*	*EF1B*	*UBC35*	*LOS1*	*ACT2*	*HAF1*
Comprehensive	*UBQ9*	*GAPDH*	*ACT1*	*UEP*	*ALDH*	*UBC35*	*EF1B*	*LOS1*	*ACT2*	*HAF1*
**C. Cold stress (Ranking order from better-good-average)**										
geNorm	*UBC35/ACT2*	*EF1B*	*GAPDH*	*UBQ9*	*UEP*	*LOS1*	*ALDH*	*ACT1*	*HAF1*	
NormFinder	*GAPDH*	*EF1B*	*UBC35*	*ACT2*	*UBQ9*	*LOS1*	*UEP*	*ALDH*	*ACT1*	*HAF1*
Bestkeeper	*GAPDH*	*EF1B*	*ALDH*	*LOS1*	*UBC35*	*ACT1*	*ACT2*	*UBQ9*	*UEP*	*HAF1*
∆CT	*GAPDH*	*EF1B*	*UBC35*	*ACT2*	*UBQ9*	*LOS1*	*UEP*	*ALDH*	*ACT1*	*HAF1*
Comprehensive	*UBC35*	*GAPDH*	*EF1B*	*ACT2*	*UBQ9*	*UEP*	*LOS1*	*ALDH*	*ACT1*	*HAF1*
**D. Salt stress (Ranking order from better-good-average)**										
geNorm	*ACT2/UEP*	*GAPDH*	*ACT1*	*EF1B*	*LOS1*	*HAF1*	*UBC35*	*ALDH*	*UBQ9*	
NormFinder	*GAPDH*	*ACT1*	*HAF1*	*ACT2*	*UEP*	*LOS1*	*UBC35*	*EF1B*	*UBQ9*	*ALDH*
Bestkeeper	*GAPDH*	*ACT1*	*LOS1*	*UBQ9*	*EF1B*	*UEP*	*HAF1*	*ACT2*	*UBC35*	*ALDH*
∆CT	*GAPDH*	*ACT1*	*HAF1*	*ACT2*	*UEP*	*LOS1*	*UBC35*	*EF1B*	*ALDH*	*UBQ9*
Comprehensive	*GAPDH*	*ACT1*	*ACT2*	*UEP*	*HAF1*	*UBC35*	*LOS1*	*EF1B*	*ALDH*	*UBQ9*
**E. Tissues (Ranking order from better-good-average)**										
geNorm	*ACT1/UBQ9*	*GAPDH*	*UEP*	*ALDH*	*EF1B*	*UBC35*	*ACT2*	*HAF1*	*LOS1*	
NormFinder	*UBC35*	*GAPDH*	*ACT1*	*UEP*	*UBQ9*	*EF1B*	*ACT2*	*ALDH*	*HAF1*	*LOS1*
Bestkeeper	*UBC35*	*UEP*	*ACT1*	*UBQ9*	*GAPDH*	*HAF1*	*ALDH*	*EF1B*	*ACT2*	*LOS1*
∆CT	*ACT1*	*GAPDH*	*UBQ9*	*UEP*	*UBC35*	*EF1B*	*ALDH*	*ACT2*	*HAF1*	*LOS1*
Comprehensive	*ACT1*	*GAPDH*	*UBC35*	*UBQ9*	*ACT2*	*UEP*	*EF1B*	*ALDH*	*HAF1*	*LOS1*
**F. All samples (Ranking order from better-good-average)**										
geNorm	*GAPDH*/*UBQ9*	*ACT1*	*UBC35*	*UEP*	*ALDH*	*EF1B*	*ACT2*	*HAF1*	*LOS1*	
NormFinder	*GAPDH*	*UBQ9*	*UBC35*	*UEP*	*ACT1*	*EF1B*	*ACT2*	*HAF1*	*ALDH*	*LOS1*
Bestkeeper	*GAPDH*	*UBQ9*	*UBC35*	*ACT2*	*UEP*	*EF1B*	*HAF1*	*LOS1*	*ACT1*	*ALDH*
∆CT	*GAPDH*	*UBQ9*	*UBC35*	*UEP*	*ACT1*	*EF1B*	*ACT2*	*ALDH*	*HAF1*	*LOS1*
Comprehensive	*GAPDH*	*UBQ9*	*UBC35*	*UEP*	*ACT1*	*EF1B*	*ACT2*	*ALDH*	*HAF1*	*LOS1*

### Reference gene validation

**Figure 4:**
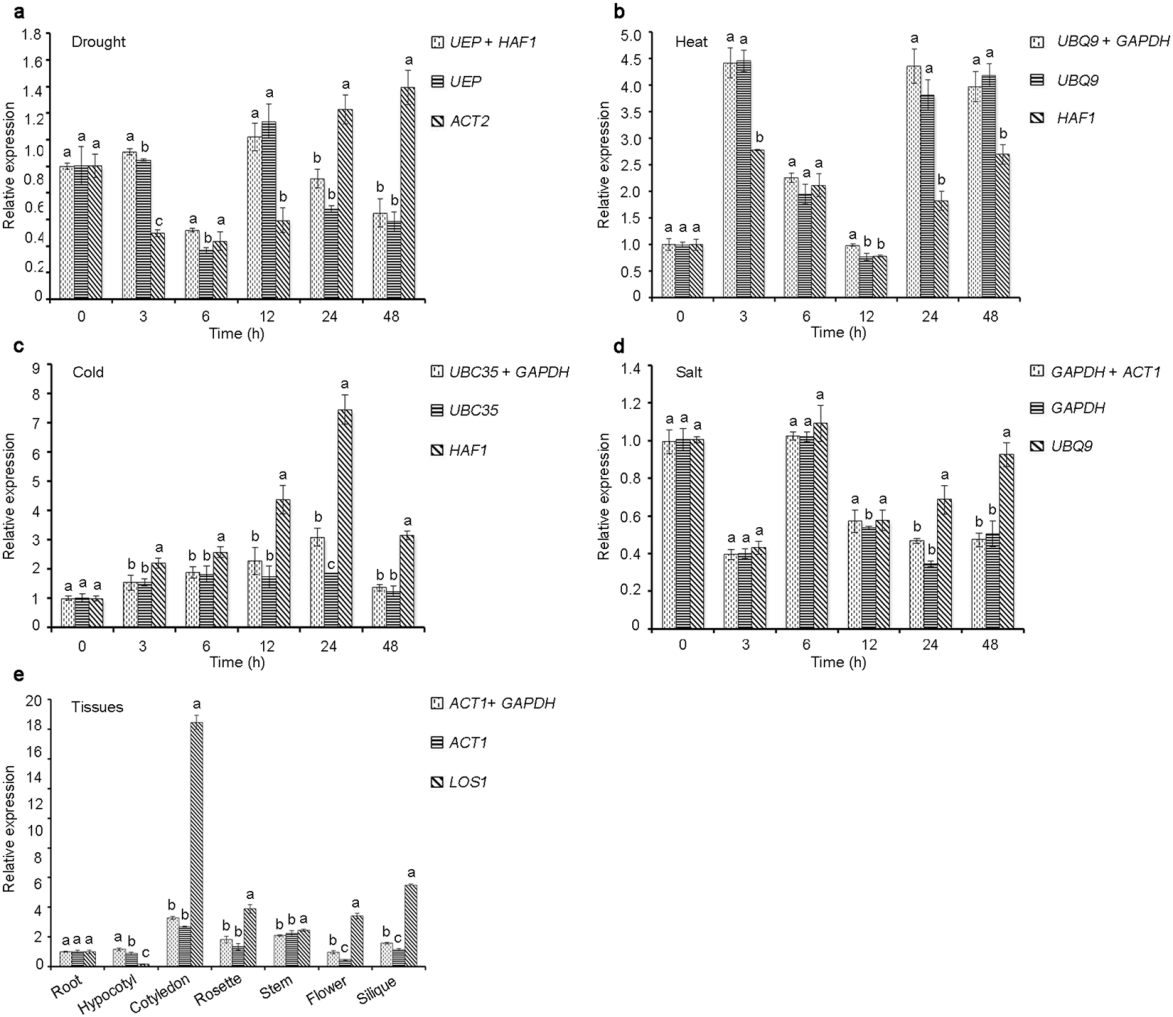
Relative expression levels of *KUP9* across all experimental sets normalized by the most stable reference gene combination, the most stable gene and the most unstable gene. (**a**) Drought stress. (**b**) Heat stress. (**c**) Cold stress (**d**) Salt stress. (**e**) Tissues. Error bars represent the standard deviation of three biological replicates. Different lowercase letters represent statistically significant differences as determined by one-way ANOVA (*P* < 0.05, Duncan’s multiple range tests).

The K^+^ uptake permease 9 (*KUP9*) gene, which functions in the potassium ion transporter, was used for reference gene validation in this study. As shown in Fig. 4, when the best reference gene combinations and the most stable genes were used for normalization analysis, the expression profiles of *KUP9* obtained through qRT-PCR were similar in all experimental groups. Under drought stress conditions, *KUP9* expression fluctuated significantly (Fig. 4a). The expression of *KUP9* was downregulated after 6 h of drought stress, upregulated quickly at 12 h and was then continuously downregulated. However, *KUP9* expression began to increase continuously after 12 h of drought stress, when the least stable gene was selected as the reference gene. When the optimal internal reference gene combination *UEP* and *HAF1* and the most stable internal reference gene *UEP* were used to normalize the relative expression changes of *KUP9*, the expression changes of *KUP9* were basically the same, especially at 12 h and later (*P* > 0.05), but when the least stable reference gene *ACT2* was used, the expression of *KUP9* was significantly changed compared with the former two results (*P* < 0.05) (Fig. 4a). Under heat stress conditions, when the most stable genes, i.e., the *UBQ9* and *GAPDH* reference gene combination, were used for normalization, the expression level of *KUP9* rose sharply to the highest point (approximately 4.5-fold higher than the control group) after 3 h of stress, then gradually decreased to the level of the control group from 6 h to 12 h, and finally *KUP9* showed a high level of expression at 24–48 h of stress (Fig. 4b). Furthermore, when the most stable gene, *UBQ9*, was used as an internal reference, the expression trend of *KUP9* was basically consistent with the previous analysis. However, when the most unstable gene, *HAF1*, was used as a reference gene, the expression of *KUP9* did not show a clear expression trend. For cold stress, when the best reference gene combination, *UBC35* and *GAPDH*, was used for normalization, *KUP9* expression only increased slightly and the same result also occurred with the most stable reference gene *UBC35* (Fig. 4c). When the least stable gene *HAF1* was used for normalization, a sustained increase in the expression level of *KUP9* was observed after stress, and a peak point was observed at 24 h, which clearly exhibited an overestimation of expressionand compared with the results of the expression changes of the former two, the relative expression of *KUP9* has changed significantly (*P* < 0.05).

Under salt stress, the expression of *KUP9* first decreased, and after increasing at 6 h, decreased again (Fig. 4d). *KUP9* showed consistent expression profiles when a combination of reference genes (*GAPDH* and *ACT1*) or the single most stable gene (*GAPDH*) was used to normalize the expression. However, when *UBQ9* (unstable gene) was used for normalization, *KUP9* expression was significantly affected at 24 h and 48 h of salt stress (Fig. 4d). Similarly, *KUP9* showed similar expression patterns when a combination of the most stable reference genes (*ACT1* and *GAPDH*) or the single most stable gene (*ACT1*) was used to normalize the expression (Fig. 4e) in different tissues. The expression level of *KUP9* in cotyledons was over stated when the most unstable gene, *LOS1*, was used for normalization compared with the results of most reference gene and the combination of reference genes (*P* < 0.05).

## Discussion

In recent years, our group has been trying to demystify the gene expression dynamics of *A. pumila* adaptation to adversity. However, the application of qRT-PCR in *A. pumila* has been limited due to a lack of reference gene information for a variety of experimental contexts. Therefore, suitable reference genes should first be screened prior to large-scale analysis of gene expression profiles under various stress conditions and in different developmental tissues in *A. pumila*. As a result, in our analysis of potential candidate reference genes in *A. pumila*, we noted that there was no single reference gene that exhibited a constant expression level in all the samples under various experimental conditions, similar to the findings for a number of plant species such as *Arabidopsis thaliana*^17^, *Caragana intermedia*^31^, *Gossypium hirsutum*^32^, *Pennisetum glaucum*^33^.

In the present study, 10 candidate internal reference genes (*ACT1*, *ACT2*, *ALDH*, *EF1B*, *HAF1*, *GAPDH*, *LOS1*, *UBQ9*, *UBC35* and *UEP*) were identified from our previous transcriptome data^3^. The PCR results showed that each pair of primers had a specific band (Supplementary Fig. S2), and the dissolution curve had only a single peak (Supplementary Fig. S3). The amplification efficiency of the primer was between 90.3% and 99.1% (Table 2).

The stability of the candidate reference genes was calculated and analysed using four different analysis software packages, geNorm^22^, NormFinder^14^, Bestkeeper^23^ and RefFinder^24,25^, to evaluate the best reference genes among 93 samples under different experimental conditions and tissues. We noted that the 10 reference genes exhibited variable expression stability in response to different stresses. For example, under drought stress conditions, geNorm software ranked *GAPDH*/*ALDH* as the best reference genes (Fig. 2a), and NormFinder determined *UEP* as the best reference gene (Table 3). BestKeeper regarded *LOS1* as the most stable reference gene (Table 4), and ΔCt inferred that *UEP* was the most stable reference gene according to its lowest mean SD value (Supplementary Table S2). Under heat stress, *UBQ9* and *GAPDH* were ranked as the top one and two positions by NormFinder and ΔCt, respectively. GeNorm ranked *ACT1* and *GAPDH* as the most stable and *UBQ9* as the second most stable. Both NormFinder and Bestkeeper ranked *UBQ9* as the most stable gene. As a result, we inferred that *UBQ9* and *GAPDH* are the best combination of stable reference genes for qRT-PCR in response to heat stress. Based on the above results, we found that it was difficult to determine stable reference genes in *A. pumila* using only one programme. Therefore, we comprehensively evaluated the results of all reference gene analyses by RefFinder and finally determined the stability orders for all reference genes under different experimental conditions.

Ubiquitin is a small regulatory protein found in all eukaryotes^34^. Genes encoding ubiquitin-like proteins that have a remarkably conserved gene structure among higher plants, animals and fungi are often used as reference genes for normalization in many reports. According to the RefFinder analysis, we found that *UEP*, *UBQ9*, and *UBC35* were the most stable reference genes under drought stress, heat stress and cold stress, respectively (Table 5). Although the most stable reference gene or reference gene combinations showed differences under different stresses, *GAPDH* exhibited more stable expression under all abiotic stresses. For example, under salt treatment, *GAPDH* was ranked in the top position by NormFinder, Bestkeeper and RefFinder. GeNorm determined that *GAPDH* was the most stable reference gene under drought stress and heat stress. Similarly, *GAPDH* was the best reference gene under cold stress according to NormFinder, Bestkeeper and RefFinder.

*ACT* has been considered to be the reference gene in some plants in response to drought and salt stress^35,36^ and in different tissues^37^. However, in the present study, *ACT2* was ranked in the last position according to the comprehensive ranking results under drought stress (Table 5). When *ACT2* was used as a reference gene to detect the expression pattern of *KUP9* under drought stress, obviously different expression profiles appeared (Fig. 4).

*KUP9* encoding a potassium ion transmembrane transporter was used as a target gene to verify the stability of the reference genes under various stress conditions. *A. thaliana AtKUP9* is expressed most highly in young leaves and can be regulated by potassium (K^+^)^38^. Our experiments indicated that the expression of the *A. pumila KUP9* gene changed significantly under various stress conditions, and its molecular mechanisms of stress tolerance deserve further study. Validation of gene expression revealed that *KUP9* showed similar expression patterns when normalized by the most stable reference gene combinations and the single most stable reference gene (Fig. 4). However, when the most unstable reference gene was used for normalization analysis, the expression level of *KUP9* was exaggerated or the expression level of *KUP9* was underestimated, suggesting that the identified reference genes are reliable.

In summary, we recommend the use of different reference genes or reference gene combinations to obtain more accurate and reliable results for qRT-PCR in *A. pumila*. Furthermore, two reference gene combinations had V_2_/_3_ values less than 0.15 across all experimental subsets (Fig. 3); consequently, *UEP* and *HAF1* under drought stress, *UBQ9* and *GAPDH* under heat stress, *UBC35* and *GAPDH* under cold stress, *GAPDH* and *ACT1* under salt stress, and *ACT1* and *GAPDH* in different tissues are suggested for the accurate normalization of target gene expression in *A. pumila*. To our knowledge, this is the first report on the identification and validation of suitable reference genes for qRT-PCR analysis in *A*. *pumila* under various abiotic stresses and developmental tissues. The identified reference genes can facilitate accurate gene expression analysis by qRT-PCR in *A. pumila* and other ephemeral species for functional genomics studies.

## Methods

### Plant materials and stress treatments

The cultivation methods and growth conditions for *A. pumila* used in this study were the same as those published in our previous reports^2,3^. For the salinity treatment, four-week-old seedlings were transferred to a 100-mL conical flask containing Hoagland’s nutrient solution for a whole day and were then moved to a fresh Hoagland’s nutrient solution containing 250 mM NaCl. For drought treatments, 10% PEG-6000 (w/v, Shanpu, Shanghai, China) was used to water the four-week-old seedlings. For cold and heat treatments, the four-week-old seedlings were placed in an illumination incubator at 4 °C and 40 °C, respectively. Leaf samples of each individual were carefully collected at 0, 3, 6, 12, 24 and 48 h after treatments.

Tissue samples of *A. pumila* were collected at different development stages. Roots, hypocotyls and cotyledons were collected from two-week-old seedlings. Rosette leaves and stems were sampled from the vegetative growth phase (six weeks after planting). Flowers and young siliques were collected at the maturity stage (three months after planting). All collected samples were immediately frozen in liquid nitrogen and were stored at −80 °C for follow-up experiments.

### RNA isolation and cDNA synthesis

The total RNA for each sample was extracted with the RNAprep Pure Plant Kit (Tiangen Biotech, Beijing, China) according to the manufacturer’s protocol. RNA was further purified using RNase-free DNase (TIANGEN) according to the manufacturer’s guidelines. The quality, quantity and integrity of the RNA samples were assessed as described previously^3^. cDNA was synthesized from 300 ng RNA using the PrimeScript II First Strand cDNA Synthesis Kit MIX (Bioteke, Beijing, China) with oligo (dT) primers in a final volume of 20 μL according to the manufacturer’s instructions.

### Selection of candidate reference genes

Due to a lack of *A. pumila* genome information, we generated a full-length transcriptome of leaf tissues for this species using SMRT carried out on a Pacific Biosciences (PacBio, Menlo Park, CA, USA) sequencing platform^3^. Based on these transcriptome datasets, 10 reference genes with relatively stable expression (based on their FPKM and fold change values) were selected to screen the most reliable reference genes for target gene expression via qRT-PCR experiments (Table 1).

### Primer design and amplification efficiency analysis for qRT-PCR

Gene-specific primers for qRT-PCR analysis were designed using the primer 3.0 online tool (http://bioinfo.ut.ee/primer3/) according to the sequences of 10 candidate reference genes and a target gene. Primers were synthesized by the Beijing Genomics Institute (Beijing, China) with the following parameters: Tm values ranging from 50 to 65 °C, GC percent of 45–55%, primer lengths of 17–25 bp and product lengths of 80–300 bp (Table 2).

To detect the specificity and amplification efficiency of each pair of primers that we designed, we first performed RT-PCR in a 20-μL system using a Mastercycler nexus GSX1 PCR apparatus (Eppendorf AG, Hamburg, Germany) containing 60 ng of the synthesized cDNA, 2 μL rTaq buffer (TakaRa, Dalian, China), 0.2 μM of each of the primers, 200 μM each dNTP and 1 U rTaq (TakaRa). The RT-PCR amplification programme consisted of 2 min of initial denaturation at 94 °C, followed by 30 cycles of 30 s at 94 °C, 45 s at 62 °C, 30 s at 72 °C, and a final 5-min extension at 72 °C. The amplification products were evaluated using 2.5% (w/v) agarose gel electrophoresis, and the PCR products were sequenced to validate primer specificity.

qRT-PCR was carried out with the SYBR Green PCR Master Mix system (CWBIO, Beijing, China) on an Applied Biosystems 7500/7500 Fast Real-time PCR System (ABI, Foster City, CA, USA). The PCR amplification system and programme were performed as described previously^2^. Three biological duplications were performed with independently isolated RNA in all qRT-PCR. Relative gene expression levels were calculated using the 2^−ΔΔCt^ method^39^.

### Determination and validation of expression stability of reference genes

The average Ct values calculated from qRT-PCR data for all samples or genes were used for further analyses. Four different types of statistical software, geNorm^22^, NormFinder^14^, BestKeeper^23^ and RefFinder^24,25^, were applied to evaluate the expression stability of the reference genes across all experimental sets.

The geNorm programme calculates the gene expression stability value (M value) and ranks the reference genes^22^. This programme also evaluates the pairwise variation (V_n_/V_n+1_) to determine the optimal number of genes required for accurate normalization of qRT-PCR data. NormFinder software evaluates the expression stability of each gene through an assessment of within-and between-group variations using a variance analysis-based approach^14^. A higher stable value calculated by geNorm or NormFinder indicates a more unstable gene, whereas the lower the stable value, the better the stability of the gene. The Ct value of each gene was input into BestKeeper software to calculate the correlation coefficient (r), standard deviation (SD) and coefficient of variation (CV) of each sample across five experimental sets. The closer the r value is to 1, the more stable the gene expression. The comparative ΔCt analysis was assessed via the commonly used RefFinder online software (http://150.216.56.64/referencegene.php?type=reference). Furthermore, we used RefFinder to comprehensively rank the expression stability by analysing the geometric mean of Ct values of all candidate reference genes^26,40^.

### Validation of reference genes

To verify the results of our experiments, the combination of the top two best reference genes, the most stable and most unstable reference genes were used to normalize the expression of one target gene, *KUP9*, under different experimental conditions and in different tissues. The qRT-PCR experimental methods were the same as those above. Statistical significance analysis was performed using IBM SPSS statistics 19.0 (https://www.ibm.com/products/spss-statistics) based on one-way ANOVA and further evaluated using Duncan’s multiple comparison (*P* < 0.05)^3^.

## Supplementary information

Identification of reliable reference genes for qRT-PCR in ephemeral plant Arabidopsis pumila based on full-length transcriptome data
