# Supplementary material for: Identification of reliable reference genes for qRT-PCR in the ephemeral plant Arabidopsis pumila based on full-length transcriptome data
Source: Sci Rep. 2019 Jun 10;9:8408. doi: 10.1038/s41598-019-44849-1 (PMC6557819; doi:10.1038/s41598-019-44849-1)
Supplement: Supplementary file 1 — Identification of reliable reference genes for qRT-PCR in ephemeral plant Arabidopsis pumila based on full-length transcriptome data [file 41598_2019_44849_MOESM1_ESM.docx]

**Identification of reliable reference genes for qRT-PCR in ephemeral plant *Arabidopsis pumila* based on full-length transcriptome data**

Yuhuan Jin^1^, Fang Liu^1, 2^, Wei Huang^1^, Qi Sun^1^, Xianzhong Huang^1^*

^1^Special Plant Genomics Laboratory, College of Life Sciences, Shihezi University, Shihezi, Xinjiang, 832000, China

^2^State Key Laboratory of Plant Physiology and Biochemistry, College of Biological Sciences, China Agricultural University, Beijing, 100193, China

*corresponding author: [xianzhongh106@163.com](mailto:xianzhongh106@163.com)

**Supplementary Information**

Supplementary Figure S1. The ephemeral plants *Arabidopsis pumila* growing in the desert regions of Northern Xinjiang, China. In the picture, the plants with yellow flowers in the red boxes are *Arabidopsis pumila*.

Supplementary Figure S2. PCR amplifications and agarose gel electrophoresis of the 10 candidate reference genes.

Supplementary Figure S3. Dissociation curves for the 10 candidate reference genes and a target gene *KUP9* in *A. pumila*.

Supplementary Table S1. Means and standard deviations of Ct values for the 10 candidate internal reference genes calculated from qRT-PCR analysis.

Supplementary Table S2. Candidate reference genes stability analyzed by RefFinder according to the average standard deviation (SD) of Ct between each gene and the rest of them within each treatment.


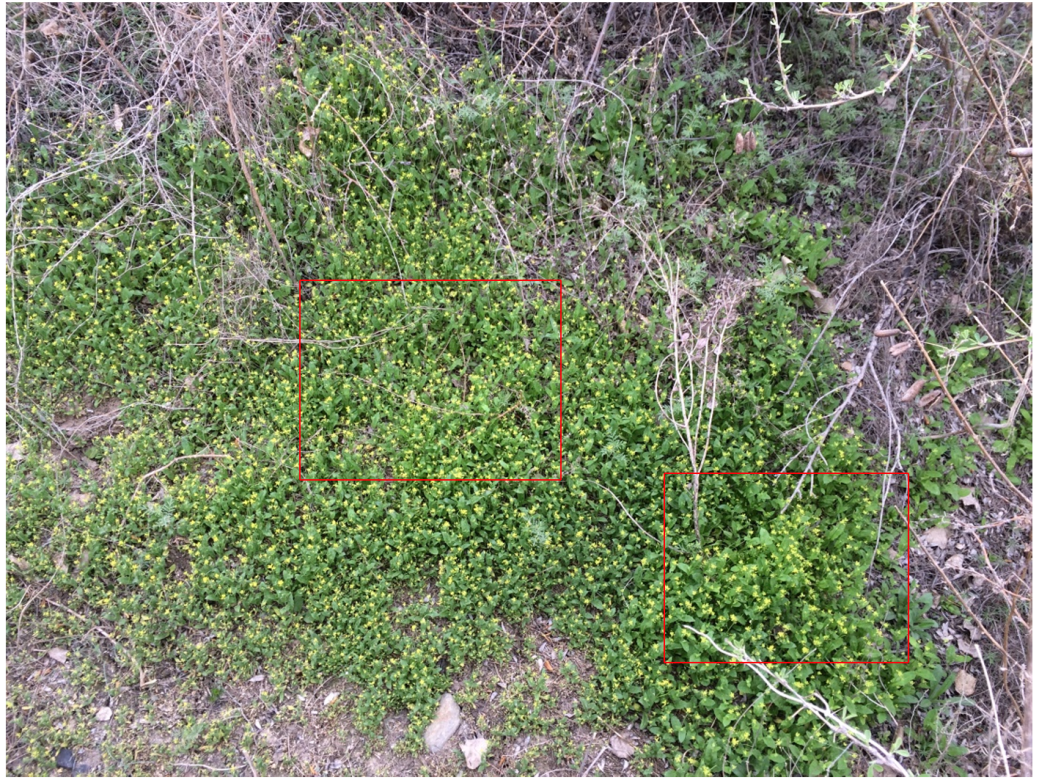


**Supplementary Figure S1. The ephemeral plants *Arabidopsis pumila* growing in the desert regions of Northern Xinjiang, China.** **In the picture, the plants with yellow flowers in the red boxes are *Arabidopsis pumila*.**


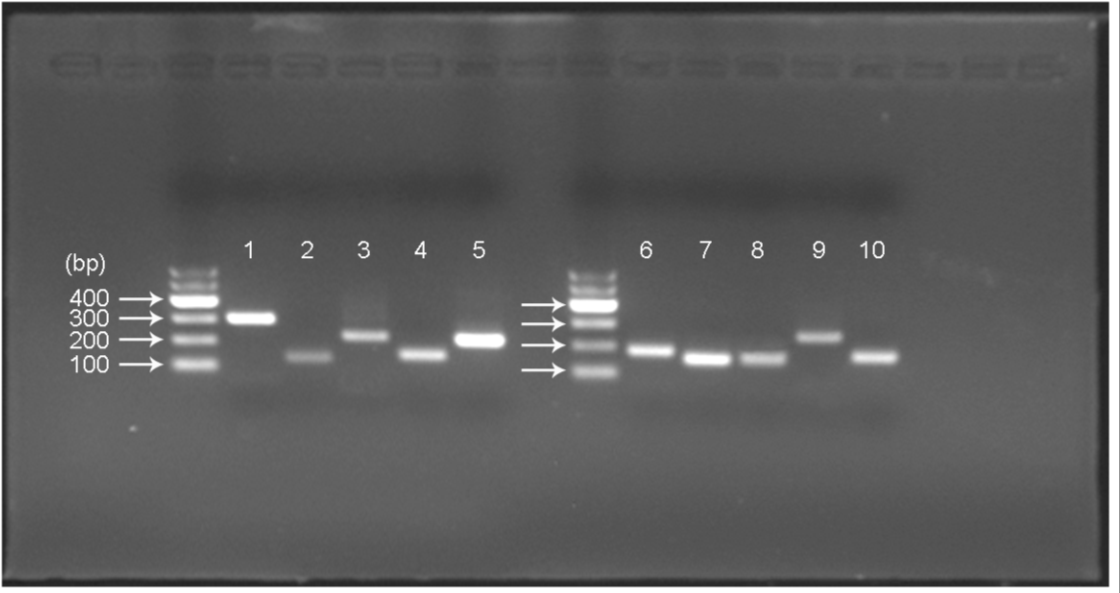


**Supplementary Figure S2. PCR amplifications and agarose gel electrophoresis of the 10 candidate reference genes.**

Lanes 1, 2, 3, 4, 5, 6, 7, 8, 9 and 10 were the gene products of *ACT1*, *ACT2*, *ALDH*, *EF1B*, *GAPDH*, *HAF1*, *LOS1*, *UBC3*5, *UBQ9* and *UEP*, respectively.


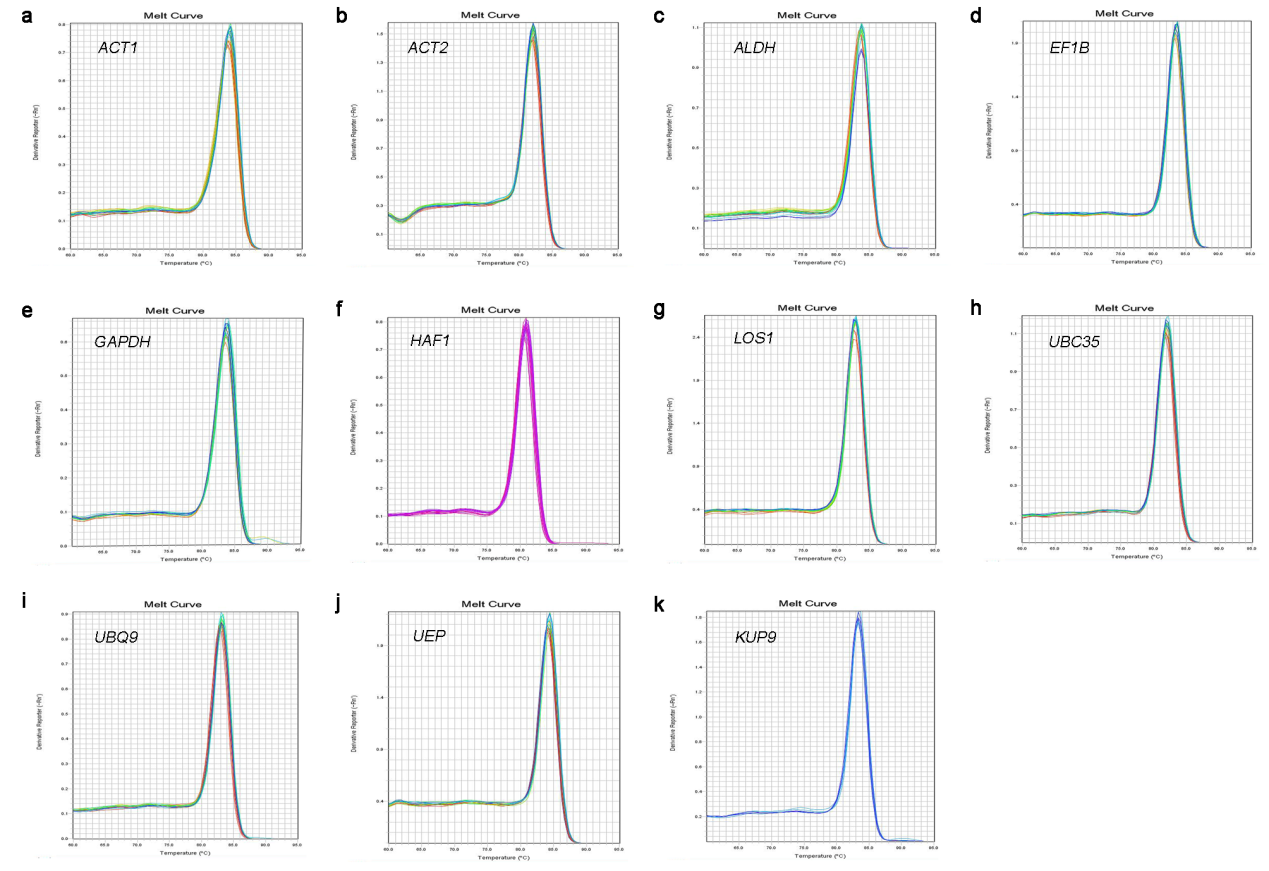


**Supplementary Figure S3. Dissociation curves for the 10 candidate reference genes and a target gene *KUP9* in *A. pumila*.**

Each peak map represents the melting curve of one gene, the peaks representing the melting temperature of each gene, and the single peak indicating no primer dimer or miscellaneous peak observed. a: *ACT1*, b: *ACT2*, c: *ALDH*, d: *EF1B*, e: *GAPDH*, f: *HAF1*, g: *LOS1*, h: *UBC35*, i: *UBQ9*, j: *UEP*, k: *KUP9*.

**Supplementary Table S1. Means and standard deviations of Ct values for the 10 candidate internal reference genes calculated from qRT-PCR analysis.**

| Genes | Drought | Heat | Cold | Salt | Tissues | All samples |
| --- | --- | --- | --- | --- | --- | --- |
| *ACT1* | 27.63 ± 1.47 | 28.57 ± 1.30 | 30.47 ± 1.38 | 27.90 ± 1.12 | 27.01 ± 2.42 | 28.79 ± 1.77 |
| *ACT2* | 22.54 ± 1.06 | 20.84 ±1.24 | 22.17 ± 0.79 | 22.55 ± 0.72 | 19.85 ± 1.95 | 22.13 ± 1.33 |
| *ALDH* | 27.49 ± 1.40 | 29.21 ± 0.97 | 30.48 ± 1.29 | 28.35 ± 0.95 | 28.87 ± 3.00 | 28.89 ± 1.61 |
| *EF1B* | 27.02 ± 1.21 | 24.74 ± 1.25 | 26.44 ± 0.69 | 25.98 ± 1.29 | 25.13 ± 2.66 | 26.45 ± 1.71 |
| *GAPDH* | 22.27 ± 1.43 | 22.73 ± 1.07 | 23.30 ± 0.93 | 22.18 ± 0.85 | 21.03 ± 2.01 | 22.63 ± 1.38 |
| *HAFI* | 27.15 ± 1.25 | 26.34 ± 1.26 | 27.75 ± 0.50 | 27.04 ± 0.77 | 26.33 ± 3.69 | 27.08 ± 1.20 |
| *LOS1* | 24.12 ± 1.40 | 25.27 ± 1.11 | 26.23 ± 1.16 | 23.85 ± 1.30 | 23.05 ± 2.30 | 24.99 ± 1.65 |
| *UBC35* | 26.56 ± 1.03 | 27.06 ± 0.94 | 28.69 ± 0.61 | 26.12 ± 0.61 | 25.63 ± 1.87 | 27.20 ± 1.31 |
| *UBQ9* | 27.34 ± 1.06 | 27.53 ± 0.86 | 28.24 ± 0.74 | 27.41 ± 1.41 | 26.37 ± 2.55 | 27.64 ± 1.25 |
| *UEP* | 22.99 ± 1.14 | 22.15 ± 0.85 | 23.06 ± 0.76 | 22.85 ± 1.05 | 20.43 ± 2.70 | 22.91 ± 1.24 |

**Supplementary Table S2. Candidate reference genes stability analyzed by RefFinder according to the average standard deviation (SD) of Ct between each gene and the rest of them within each treatment.**

| Rank | Drought | | Heat | | Cold | | Salt | | Tissues | | All samples | |
| --- | --- | --- | --- | --- | --- | --- | --- | --- | --- | --- | --- | --- |
|  | genes | Mean SD | genes | Mean SD | genes | Mean SD | genes | Mean SD | genes | Mean SD | genes | Mean SD |
| 1 | *UEP* | 0.50 | *UBQ9* | 0.69 | *GAPDH* | 0.71 | *GAPDH* | 0.61 | *ACT1* | 1.56 | *GAPDH* | 0.93 |
| 2 | *HAF1* | 0.52 | *GAPDH* | 0.77 | *EF1B* | 0.71 | *ACT1* | 0.68 | *GAPDH* | 1.62 | *UBQ9* | 0.97 |
| 3 | *LOS1* | 0.54 | *ALDH* | 0.79 | *UBC35* | 0.72 | *HAF1* | 0.70 | *UBQ9* | 1.62 | *UEP* | 1.04 |
| 4 | *UBQ9* | 0.59 | *ACT1* | 0.83 | *ACT2* | 0.77 | *ACT2* | 0.75 | *UEP* | 1.66 | *UBC35* | 1.07 |
| 5 | *ALDH* | 0.61 | *UEP* | 0.87 | *UBQ9* | 0.82 | *UEP* | 0.76 | *UBC35* | 1.69 | *LOS1* | 1.10 |
| 6 | *ACT1* | 0.63 | *EF1B* | 0.88 | *LOS1* | 0.93 | *LOS1* | 0.81 | *EF1B* | 1.98 | *HAF1* | 1.10 |
| 7 | *GAPDH* | 0.64 | *UBC35* | 0.90 | *UEP* | 0.93 | *UBC35* | 0.82 | *ALDH* | 2.06 | *ACT1* | 1.12 |
| 8 | *EF1B* | 0.65 | *LOS1* | 0.93 | *ALDH* | 0.96 | *EF1B* | 0.83 | *ACT2* | 2.23 | *ALDH* | 1.18 |
| 9 | *UBC35* | 0.68 | *ACT2* | 1.03 | *ACT1* | 1.06 | *ALDH* | 0.89 | *HAF1* | 2.79 | *ACT2* | 1.22 |
| 10 | *ACT2* | 0.74 | *HAF1* | 1.08 | *HAF1* | 1.17 | *UBQ9* | 0.90 | *LOS1* | 3.63 | *EF1B* | 1.38 |
